# Interplay of HCPro and CP in the Regulation of Potato Virus A RNA Expression and Encapsidation

**DOI:** 10.3390/v14061233

**Published:** 2022-06-07

**Authors:** Shreya Saha, Andres Lõhmus, Pinky Dutta, Maija Pollari, Kristiina Mäkinen

**Affiliations:** Department of Microbiology, Viikki Plant Science Centre, University of Helsinki, 00014 Helsinki, Finland; ss2344@cam.ac.uk (S.S.); andres.lohmus@gmail.com (A.L.); pinky.dutta@helsinki.fi (P.D.); maija.pollari@helsinki.fi (M.P.)

**Keywords:** potyvirus, potato virus A, helper-component proteinase (HCPro), coat protein (CP), viral gene expression, encapsidation, particle stability

## Abstract

Potyviral coat protein (CP) and helper component-proteinase (HCPro) play key roles in both the regulation of viral gene expression and the formation of viral particles. We investigated the interplay between CP and HCPro during these viral processes. While the endogenous HCPro and a heterologous viral suppressor of gene silencing both complemented HCPro-less potato virus A (PVA) expression, CP stabilization connected to particle formation could be complemented only by the cognate PVA HCPro. We found that HCPro relieves CP-mediated inhibition of PVA RNA expression likely by enabling HCPro-mediated sequestration of CPs to particles. We addressed the question about the role of replication in formation of PVA particles and gained evidence for encapsidation of non-replicating PVA RNA. The extreme instability of these particles substantiates the need for replication in the formation of stable particles. During replication, viral protein genome linked (VPg) becomes covalently attached to PVA RNA and can attract HCPro, cylindrical inclusion protein and host proteins. Based on the results of the current study and our previous findings we propose a model in which a large ribonucleoprotein complex formed around VPg at one end of PVA particles is essential for their integrity.

## 1. Introduction

Potyviruses form a widespread and economically important group of viruses. Their single-stranded plus-sense genomic RNA encodes eleven proteins. Ten of these proteins are processed from a large polyprotein by virus-encoded proteinases and the eleventh is produced from a reading frame nested in the protein P3 encoding region. The helper-component proteinase (HCPro) is a multifunctional potyviral protein [1]. HCPro acts as a potent antiviral silencing suppressor [2,3]. Its silencing suppression capacity enables synergistic interactions with other viruses [4,5]. Furthermore, HCPro is required for virus polyprotein processing [6], virion formation [7], systemic movement [8,9], and aphid transmission of the virus [10,11]. HCPro achieves these functions in cooperation with other viral and host proteins. Coat protein (CP) is the structural protein of potyviruses. Like HCPro, CP has many functions in the potyviral infection cycle. It is required for cell-to-cell movement [12] and aphid transmission between plants [13]. CP inhibits PVA translation in a dose-dependent manner relying on self-interactions [14,15]. Interestingly, potyviral HCPro and CP seem to have a virus-specific interaction that regulates virion formation and virus accumulation [7,16]. Plum pox virus (PPV) HCPro stabilizes its cognate CP and promotes particle accumulation: an HCPro mutation that did not hinder silencing suppression interfered with virion formation [7]. This suggests that HCPro’s capacity to promote virion accumulation is independent of its silencing suppression function. Recently, it was shown that P3 is also required for CP stabilization and virion formation in potyviruses [17].

The process of virion formation in potyvirus infections remains obscure and requires more investigation. The results in [18] demonstrate that an octameric ring-like assembly intermediate is required for the formation of pepper vein banding virus (PVBV) particles in vitro. The N- and C-terminal CP regions are crucial in this process although they can be removed after assembly without losing the stability of the PVBV particles. The high-resolution virion structures of watermelon mosaic virus, potato virus Y (PVY) and turnip mosaic virus (TuMV) were published recently [19,20,21]. These structures help to pinpoint and understand the exact RNA-CP interactions that are crucial for the helical configuration and stability of the virion. Both HCPro and the cylindrical inclusion protein (CI), a viral DExD/H–box-like RNA helicase, are associated with a tip structure at one end of potyviral particles [22,23]. Interestingly, also the plant eukaryotic initiation factor eIF4E associates with lettuce mosaic virus (LMV, potyvirus) particles via the 5′ RNA-linked VPg [24]. Whether eIF4E gets attached during the assembly of LMV particles or if it is recruited after assembly remains to be studied.

PPV RNA can be encapsidated only if it is capable of replication [17], the biological significance of which could be to assure the infectivity of the particles. We have previously proposed that PVA RNA engages in particle assembly directly from translating polysomes [14]. The results in [14] suggested that CP inhibits viral RNA (vRNA) translation through co-translational interactions between excess CP accumulated in trans and CP translated from vRNA in cis. 

In this study, we performed experiments to reveal how HCPro and CP coordinate viral gene expression and particle formation. The results further highlight the separate functions of potyviral HCPro in viral gene expression and particle formation. PVA CP has important functions associated with polysomes in the regulation of translation and replication [25]. These functions related to viral gene expression are regulated by host factors such as heat shock proteins HSP70, HSP40 and protein kinase CK2 [15,25,26]. Here, we studied the role of HCPro connected to viral protein synthesis and propose that HCpro rescues CP-mediated translational inhibition by sequestering CPs abundantly to particles. Finally, we provide evidence that replicated PVA RNA, which carries VPg, is encapsidated into stable virions with the aid of HCPro, whereas packaging of non-replicating vRNA, carrying a 5′ cap-structure, results in the formation of unstable particles. We show that these highly vulnerable particles can form in infected cells but readily break down upon extraction from plant tissues. These results combined with those from previous studies support the possibility that a protein complex around genome-linked VPg stabilizes the particle structure. The putative connection of these findings with the previously reported tip structure at one end of potyvirus particles [23] is discussed.

## 2. Materials and Methods

### 2.1. Plants and Agrobacterium Strain

*Nicotiana benthamiana* plants were used in all experiments. Plants were grown in a greenhouse in 16 h of light at 22 °C and 8 h of dark at 18 °C. Plants were infiltrated at the 4- to 6-leaf stage as described previously [27]. In infiltrations, we used *Agrobacterium tumefaciens* strain C58C1 carrying the pGV2260 helper plasmid for *vir* gene expression. 

### 2.2. Virus and Transient Protein Expression Constructs

**Figure 1:**
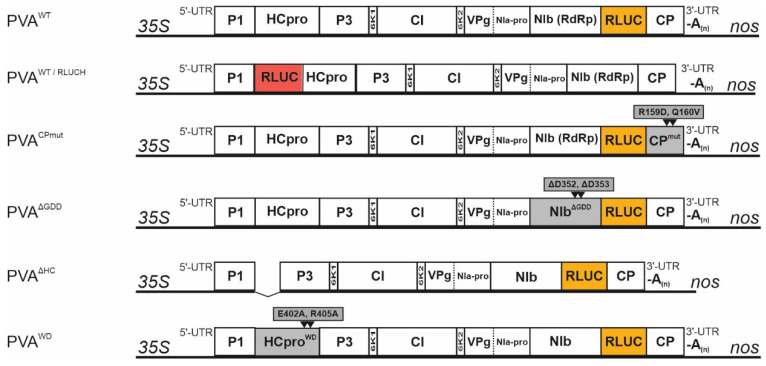
Schematic diagrams of viral constructs used in this study.

Viral constructs used in the infection experiments were based on the full-length infectious cDNA of PVA strain B11 (GenBank accession number AJ296311) [28] tagged with a *Renilla* luciferase reporter *rluc* (pro35S -PVA:*rluc*) [27]. PVA^CPmut^ and PVA^ΔGDD^ were described in [27], PVA::RLUCH in [29]. PVA^ΔHCPro^ in [30] and PVA^WD^ in [31]. All PVA constructs used in this study are overviewed in Figure 1. Transient expression constructs for individual CPs were as in [15,25]. RFP-tagged transient expression constructs for HCPro, HCPro^SD^ and HCPro^4Ebd^ were described in [30] and HCPro^WD^ in [31]. GUS and cucumoviral 2b expression constructs were described in [27,30]. The PVA CP gene was tagged with an N-terminal YFP fusion by Gateway cloning. The CP gene was PCR-amplified from the infectious complementary DNA (icDNA) of PVA B11 [28] with Gateway-compatible oligos and cloned into the pGWB42 destination vector [32] via pDONRZeo (Invitrogen, Waltham, MA, USA). 

### 2.3. Agrobacterium Infiltration of Virus and Transient Protein Expression Constructs

*Agrobacterium tumefaciens* strain C58C1 containing the viral icDNA or protein constructs was grown in Luria Broth supplemented with 10 mM morpholineethanesulfonic acid (MES) (pH 6.3), 20 μM acetosyringone, carbenicillin (100 μg/mL) and kanamycin (50 μg/mL)] overnight at 28 °C. Fresh medium was inoculated with the primary culture (1:25) and incubated at 28 °C for 5 h. The secondary culture was centrifuged at 5000× *g* for 5 min at 20 °C and washed twice with induction buffer (10 mM MES pH 6.3, 10 mM MgCl_2_ and 150 μM acetosyringone) to harvest the cells. The infiltration mixes were prepared in induction buffer and recovered for 2 to 3 h at room temperature prior to infiltration. *Agrobacterium* strains carrying virus and protein expression constructs were infiltrated alone or in combinations, at the required ratios in the final infiltration mix. Two fully developed upper leaves of *N. benthamiana* plants were infiltrated on the abaxial side. The protein expression constructs were infiltrated at *Agrobacterium* concentration OD_600_ 0.3 a day prior to infiltrating the viral icDNA constructs (OD_600_ 0.05). A firefly luciferase protein expression construct was used in the experiments as an internal control and was infiltrated (OD_600_ 0.005 or 0.001) together with the viral constructs. Exceptions to these OD_600_ values are explained in connection to the corresponding experiment.

### 2.4. Quantification of Viral Gene Expression

To measure PVA gene expression, leaf samples were collected from the inoculated leaves using a cork borer with a radius of 3 mm. The samples were immediately frozen in liquid nitrogen and crushed with six 2 mm stainless steel beads in a Mixer Mill (MM 400, Retsch, Haas, Germany) at 30 Hz for 1.5 min. The samples were prepared and the expression of the *Renilla* and firefly luciferase reporters was measured using the Dual-Luciferase Reporter Assay System (Promega, Madison, WI, USA). Half of the recommended volumes of Luciferase Assay Buffer II and Stop & Glo were used in the assay. The activity of virus-derived *Renilla* luciferase was normalized to ectopically expressed Firefly luciferase in the same samples.

### 2.5. Reverse Transcription-Quantitative PCR

We performed reverse transcription quantitative PCR (RT-qPCR) from the infected *N. benthamiana* leaf samples to quantify total PVA RNA. Leaf discs were collected at different time points as described in the results. Each sample set comprised of a pooled sample from four plants. Each RT-qPCR experiment was performed independently at least two times. The leaf samples were frozen immediately and either stored at −70 °C or ground in liquid nitrogen. Total RNA from the ground tissue was isolated using the RNeasy plant mini kit (Qiagen, Hilden, Germany) according to the manufacturer’s protocol. One microgram of total RNA was treated with RNase-free DNase I (Thermo Scientific, Waltham, MA, USA). First-strand cDNA synthesis was performed by using RevertAid H Minus first-strand cDNA synthesis kit (Thermo Scientific, Waltham, MA, USA) using random hexamers. RT-qPCR was performed from the cDNA in 96-well plates using a CFX96 Touch real-time PCR system (Bio-Rad, Hercules, CA, USA). Each reaction mixture of 10 μL in volume contained 5 μL Maxima SYBR green qPCR master mix (Thermo Scientific, Waltham, MA, USA), 0.5 μM of each forward and reverse primers, 1 μL cDNA, and 3 μL nuclease-free water. Three technical replicates were performed from each cDNA sample. The following primer pairs were used: CPqPCRF′ (5′-CATGCCCAGGTATGGTCTTC-3′)/CPqPCRR′ (5′-ATCGGAGTGGTTGCAGTGAT-3′). The housekeeping gene protein phosphatase 2A (PP2A) was used as a reference gene. PP2A was amplified with the primers described previously [33] The amplification parameters for qPCR were 3 min of initial denaturation at 95 °C, followed by 39 cycles of 10 s of denaturation at 95 °C, 30 s of annealing at 55 °C, and 30 s of synthesis at 72 °C. A melting curve was generated by heating from 60 °C to 95 °C in increments of 0.5 °C/s. The following controls were included: template replaced by nuclease-free distilled water (non-template control) and RT reaction mixtures lacking the reverse transcriptase (RT-negative controls). In order to determine the vRNA copy number serial dilutions of a PVA icDNA plasmid were used to generate a linear standard curve in the same qPCR run. Known concentrations of the plasmid template allowed the calculation of copy numbers based on Cq values.

### 2.6. Immunocapture RT-qPCR

We performed immunocapture RT-qPCR (IC-RT-qPCR) to quantitate virion-associated vRNA. Each IC-RT-qPCR experiment was performed at least twice. Leaf discs were collected at different time points as described in the results. Each sample set consisted of a pooled sample from four plants. The leaf samples were frozen immediately and either stored or ground in liquid nitrogen. The ground leaf samples for this experiment were mixed with 200 µL of sample extraction buffer [1.4 mM KH_2_PO_4_, 8 mM Na_2_HPO_4_, 136 mM NaCl, 2.6 mM KCl (pH 7.4), 0.05% Tween 20, 8 M polyvinylpyrrolidone, 0.2% BSA] and were allowed to settle on ice for 30–45 min. 96-well plates pre- coated with anti-PVA CP antibody were used to capture the CP assemblies from the infected leaf samples. For coating, PVA anti-CP antibody [PVA mix MAb; Science and Advice for Scottish Agriculture (SASA), Edinburgh, UK] was diluted 1:1000 in coating buffer [15 mM Na_2_CO_3_, 34 mM NaHCO_3_ (pH 9.6)] and incubated in the wells for 3 h at 37 °C. The plates were washed 3 to 4 times with wash buffer [1.4 mM KH_2_PO_4_, 8 mM Na_2_HPO_4_, 136 mM NaCl, 2.6 mM KCl (pH 7.4), 0.05% Tween 20] to remove unbound antibody. 100 µL of leaf lysate, suspended in extraction buffer, was added in each well of the coated plate and incubated overnight at 4 °C. A non-infected mock sample extract was used as a negative control for the experiment to determine background signal levels. Plates were washed gently with wash buffer and used for cDNA preparation and RT-qPCR as described above.

### 2.7. Confocal Microscopy

We checked the expression of HCPro^RFP^ and CP^YFP^ *in planta* by using confocal microscopy. *N. benthamiana* leaves were infiltrated with HCPro^RFP^ and CP^YFP^ at OD_600_ 0.3 in three parallel experiments. At 3 days post infiltration (dpi), four leaf discs surrounding the infiltration point were cut by a cork borer and mounted on an objective glass with a few drops of water. The abaxial side of the leaf discs was scrutinized with confocal laser scanning microscopy (Leica TCS SP5II, Leica Microsystems, Wetzlar, Germany). For visualizing CP^YFP^, excitation was performed with the argon laser at 488 nm and emission was recorded at 525–555 nm. HCPro^RFP^ was excited with a DPSS 561 nm laser and emission recorded at 570–620 nm (DD 488/561 beam splitter). Sequential scanning mode was applied for co-imaging of fluorescent proteins. The images were acquired with a 63× water immersion objective. Co-localization analysis of HCPro^RFP^ and CP^YFP^ were performed with Fiji (ImageJ) image analysis software package (version 1.51, [34]) using the co-localization threshold-function.

### 2.8. SDS-PAGE and Western Blot

Leaf samples *Agrobacterium* infiltrated with viral and protein expression constructs were harvested at 3–4 dpi to detect CP and HCPro levels. The samples were obtained and ground similarly as described above and total protein was prepared. Proteins were separated on a 12% SDS-PAGE (sodium dodecyl sulfate polyacrylamide gel electrophoresis) gel and transferred on to polyvinylidene difluoride (PVDF) membrane as per standard procedures. The membranes were blocked in 3% bovine serum albumin (BSA) dissolved in Tris-buffered saline (TBS) (50 mM Tris-HCl [pH 7.4], 150 mM NaCl) containing 0.1% Tween-20 and probed with anti-CP antiserum (1:10,000). Detection with horseradish peroxidase (HRP)-conjugated secondary antibodies (1:15,000; Promega, Madison, WI, USA) was carried out using the Immobilon Western chemiluminescent HRP substrate (Millipore, Burlington, VT, USA). A Rubisco large subunit band stained with Ponceau S was used as loading control.

### 2.9. CP Stability Assay

Samples were collected from leaves *Agrobacterium* infiltrated with viral and protein expression constructs and ground as described above. 1 mL of 0.1 M sodium phosphate buffer (pH 7.5) was added to the powdered samples. After vigorous mixing, the samples were centrifuged at 14,000× *g* for 3 min at room temperature. A sample at time point zero was obtained immediately after the centrifugation step and Laemmli sample buffer was added followed by boiling for 5 min. The remaining cell lysate was incubated at room temperature and a 60 min sample was obtained. The prepared samples were analyzed by Western blot with anti-CP antibodies. Each stability assay was performed at least two times.

### 2.10. Tissue Fixation

Infected *N. benthamina* leaf tissues were treated with formaldehyde to chemically fix any virions present in the cells. Plants were infiltrated with four different *Agrobacterium* combinations carrying protein expression and viral constructs. PVA^WT^ was infiltrated at OD_600_ 0.1, PVA^ΔGDD^ at OD_600_ 0.2 and PVA^WD^ at OD_600_ 0.2. CP and HCPro were infiltrated at OD_600_ 0.3. *Agrobacterium* harbouring PVA^ΔGDD^ and PVA^WD^ were used in double concentration to ensure adequate viral gene expression. At 4 dpi, 4 leaf discs were collected by using a 3 mm cork borer surrounding the infected area. Two sets of samples were collected from the same leaves, a control set before and an experimental set after fixing the tissue. Fixing was carried out at room temperature by infiltrating and incubating the tissues for 10 min in 0.1% formaldehyde dissolved in water (*v*/*v*), followed by infiltration and incubation of 10 min in 125 mM glycine dissolved in phosphate saline buffer to quench the reaction. Reversion of the cross-linking was performed to allow uncoating of the vRNA from the virions in the fixed tissues by keeping the samples at 70 °C for 1 h.

### 2.11. Transmission Electron Microscopy of Virus Particles

Fixed leaf samples were examined by electron microscopy (EM) for the presence of PVA particles. The tissues were ground in liquid nitrogen and mixed with 100 μL of 0.6 M sodium phosphate buffer (pH 7.4). The samples were incubated on ice for 1 h to clear the lysate. Carbon-coated electron microscopy grids were incubated with anti-CP antibodies diluted in 0.6 M sodium phosphate buffer (pH 7.4) at 1:100, (PVA mix MAb; Science and Advice for Scottish Agriculture [SASA], Edinburgh, UK), for 1 h at room temperature. Excess antibody was removed by washing twice with the same sodium phosphate buffer. Antibody-coated grids were placed on a drop of cleared leaf extracts, carefully taken without disturbing the debris. The grids were incubated at 4 °C overnight, washed with 20 drops of sodium phosphate buffer and immediately stained with 2% uranyl acetate for 12–15 s. Excess stain was drenched from the grids by using filter paper, and dried grids were used for the visualization of the particles with a Jeol JEM-1400 transmission electron microscope (Jeol Ltd., Tokyo, Japan).

### 2.12. Transmission Electron Microscopy of Leaf Sections

Virus particles in intact leaves were visualized by transmission electron microscopy of thin sections of glutaraldehyde-fixed samples. Leaf samples obtained with a 5 mm cork borer were vacuum-infiltrated for 5 min in 0.1 M Na-phosphate buffer (pH 8) containing 2.5% glutaraldehyde. The fixation was allowed to continue for 2 h at room temperature. Excess glutaraldehyde was removed by two rinses in 0.1 M sodium-phosphate buffer. Samples were embedded, cut into sections, and negatively stained at the Institute of Biotechnology Electron Microscopy Unit essentially as in [35]. The stained sections were observed with Jeol-JEM 1400 instrument (Jeol Ltd., Tokyo, Japan).

## 3. Results

### 3.1. PVA HCPro Does Not Stabilize PVA CP When Expressed in the Absence of Infection

**Figure 2:**
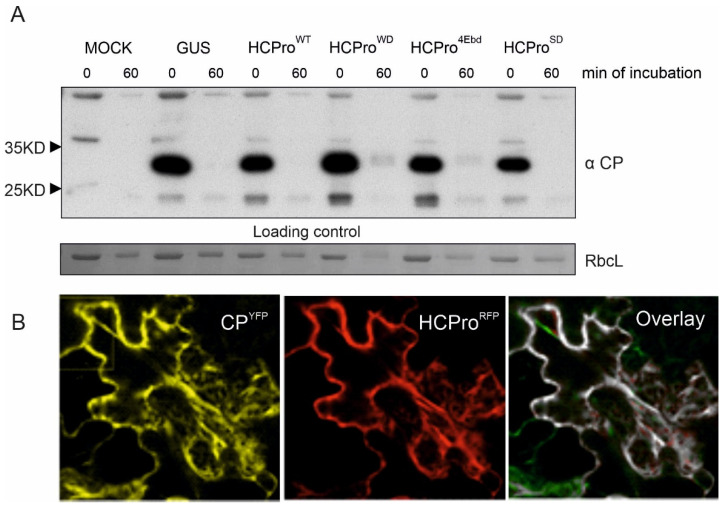
PVA HCPro alone does not increase CP stability in plant tissue lysate. (**A**) PVA CP was co-expressed with RFP-tagged versions of either PVA HCPro, HCPro^WD^, HCPro^4Ebd^ or HCPro^SD^, and the samples for western blot analysis were collected at 4 dpi. Neither HCPro nor its mutants were able to stabilize PVA CP in the in vitro degradation assay. Below is the loading control, Rubisco large subunit (RbcL) band stained with Ponceau S. (**B**) Both PVA HCPro^RFP^ and CP^YFP^ localized all over the cytoplasm when they were co-expressed. Given is also the overlay of the CP^YFP^ and HCPro^RFP^ fluorescence.

PPV HCPro provided stability for CP when infected leaf sap was subjected to an in vitro degradation assay [7]. Our first question was whether HCPro of PVA stabilizes CP in the absence of infection. We expressed either PVA HCPro or its mutants with CP by infiltrating *Agrobacterium* carrying the expression constructs into *N. benthamiana* leaves at OD_600_ 0.3. HCPro^WD^ carries a mutation in a site predicted to interact with WD40-domain proteins [31]. This mutation reduces its association with VARICOSE (VCS), an important infectivity factor for PVA. VCS participates in PVA translation together with VPg [30]. HCPro^4Ebd^ is a mutant deficient in eIF4E binding [36] and HCPro^SD^ is an RNA silencing suppression-deficient mutant [30]. Leaf samples collected at 3 dpi were subjected to a similar degradation assay as in [7,17]. This assay is based on incubation of leaf cell lysate at room temperature for an hour. A subsequent western blot analysis with anti-CP antibodies revealed that CP was degraded in all samples, showing that neither HCPro nor any of the mutants were able to stabilize CP per se (Figure 2A). To check the expression of both proteins inside the same cell, CP expression construct tagged with YFP (CP^YFP^) and HCPro expression construct tagged with RFP (HCPro^RFP^) were co- infiltrated into *N. benthamiana* plants without the virus. The expression of these proteins was checked by confocal microscopy. The result showed that both proteins were expressed in the cytoplasm (Figure 2B). Based on this result, we next investigated if PVA CP stabilization requires additional viral factors.

### 3.2. Several Viral RNA Silencing Suppressors Complement HCPro-Less PVA Gene Expression, but Only PVA HCPro Stabilizes CP in the Infection Context

**Figure 3:**
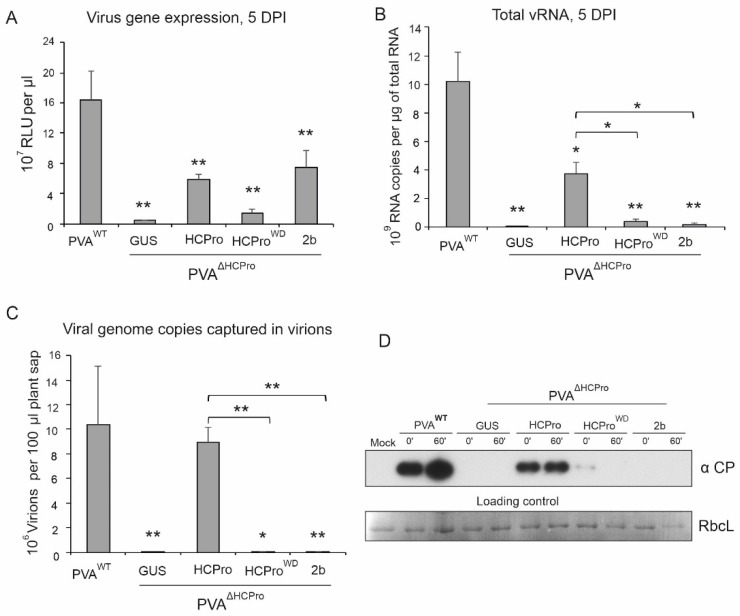
Transiently expressed HCPro complements gene expression and virion formation of HCPro-less PVA. (**A**) The RLUC activity was measured from PVA^ΔHCPro^ co-expressed with the GUS control, HCPro, HCPro^WD^ or cucumoviral suppressor of RNA silencing, 2b. (**B**) Total RNA of PVA^ΔHCPro^ in the same experiment as in (**A**). (**C**) Quantitation of PVA^ΔHCPro^ RNA by RT-qPCR of immuno-captured templates in the same experiment as in (**A**). All the samples in (**A**–**C**) were collected at 5 dpi. RLUC, total RNA and particle-associated RNA levels from PVA^WT^ infected plants is shown for comparison. (**D**) Samples from this experiment were subjected to in vitro degradation assay. A western blot analysis with anti-CP antibodies of the samples as they were at 0 min time point and after 60 min incubation of the plant sap at room temperature. RbcL band in Ponceau S-stained membrane is given as a loading control. *p* < 0.05 = *; *p* < 0.01 = **.

HCPro can complement potyvirus amplification and movement in trans [3]. We have shown earlier that HCPro supplied in trans efficiently complements the lack of endogenous HCPro also in a PVA infection [30]. In the next experiment, we expressed the HCPro-less virus (PVA^ΔHCPro^) and provided HCPro by transient expression. The gene expression of PVA is reported by the activity of *Renilla* luciferase (*Rluc*) gene located in between NIb and CP cistrons [27]. The experiment was initiated by infiltrating *Agrobacterium* carrying HCPro expression construct and followed by infiltration of the PVA^ΔHCPro^ construct to the same leaves a day later. In accordance with our previous observations, expression of PVA HCPro in-trans with PVA^ΔHCPro^ RNA increased RLUC expression from the vRNA (Figure 2A). Viral silencing suppressors (VRSs) from other virus groups [37] can support potyviral gene expression. Cucumoviral 2b, tombusviral P19 and potexviral P25 all complemented gene expression of PVA^ΔHCPro^ to varying degrees [30]. In this study, we confirmed that 2b can complement the gene expression of PVA^ΔHCPro^ with similar efficiency as HCPro (Figure 3A). HCPro^WD^, which is impaired in RNA silencing suppression [31], complemented PVA gene expression less efficiently than HCPro, as expected. The vRNA amounts in these samples were determined by RT-qPCR. RLUC accumulation and vRNA amounts from PVA^WT^ and HCPro-supplemented PVA^ΔHCPro^ were equivalent suggesting full complementation (Figure 3, compare A and B). The poor ability of HCPro^WD^ to complement PVA^ΔHCPro^ gene expression is reflected in the correspondingly low increase in PVA^ΔHCPro^ vRNA levels. Interestingly, the RLUC expression and vRNA accumulation levels did not correlate when 2b was substituted for HCPro (Figure 3, compare A and B). Similar RLUC activities were measured with both 2b and HCPro but the amount of vRNA was significantly lower in the presence of 2b than HCPro. This may mean that the majority of vRNA in HCPro-containing samples was encapsidated into virions, which was not the case in the presence of 2b. To quantitate PVA particle abundance, we subjected the samples to IC-RT-qPCR. With this method, virions and virus-like particles are captured with anti-CP antibodies. RT-qPCR is then used to measure the amount of encapsidated vRNA, which represents particle copy number. The result revealed that HCPro alone was able to support particle formation (Figure 3C).

A western blot analysis of the same samples as described above revealed an equally strong CP accumulation in the presence of HCPro, regardless of whether HCPro was produced endogenously or exogenously (Figure 3D). In the presence of GUS or 2b, CP accumulation was below the detection limit even though 2b complemented viral gene expression as well as HCPro, as assessed by RLUC activity (Figure 3D). This suggests that 2b is not able to stabilize PVA CP as effectively as HCPro. The HCPro^WD^ mutant protein was able to support low but detectable CP accumulation (Figure 3D). Next, samples from the complementation test were subjected to an in vitro degradation assay. In contrast to transiently expressed CP, CP expression from vRNA led to its stabilization when HCPro was available (compare Figure 2 and Figure 3D). A comparison of the results obtained in the presence of transiently expressed HCPro or 2b shows that while the complementation of the viral gene expression and protein production is a general silencing suppression-mediated effect, PVA CP stabilization is a separate and specialized function of HCPro. According to the results described above this function appears to be closely linked to particle formation.

### 3.3. HCPro Relieves CP-Mediated Block in Viral Gene Expression and Allows Simultaneous Encapsidation

Next, we wanted to approach the issues of initiating virion formation by examining CP-mediated translation inhibition and HCPro’s ability to influence it.

**Figure 4:**
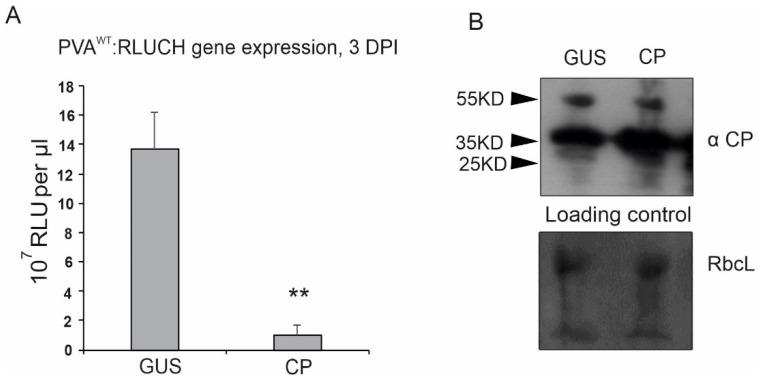
PVA CP inhibits viral RNA translation from the entire length of PVA RNA. (**A**) Viral translation from PVA^WT^ carrying the *rluc* cistron in front of the HCPro cistron (PVA^WT^:RLUCH) was determined as RLUC activity at 3 dpi. (**B**) The western blot analyses with anti-CP antibodies show CP production from the PVA genome in the GUS control lane and its transient overexpression in the CP lane. Notably, PVA RNA translation is blocked in the latter case. *p* < 0.01 = **.

The analysis was initiated with an inhibition assay. Although, it would be logical to assume that the factors that regulate viral translation affect the production of all parts of the polyprotein equally, we recently found that overexpression of VPg with vRNA increased RLUC and CP accumulation when these were the last two 3′ cistrons on the vRNA [29]. We previously showed that PVA CP inhibits RLUC production from PVA vRNAs carrying the *Rluc* cistron between NIb and CP cistrons [14]. Next, CP-mediated inhibition of PVA protein production was tested with a PVA^WT^ construct carrying the *Rluc* cistron in front of HCPro (PVA^WT^:RLUCH) near the 5′ end of the vRNA. This construct was *Agrobacterium* infiltrated (OD_600_ 0.05) one day after the CP overexpression construct (OD_600_ 0.3). RLUC activities derived from PVA^WT^:RLUCH (5′ RLUC) were determined at 3 dpi. Significant inhibition of RLUC accumulation was observed (Figure 4), which indicated that CP blocks translation from the entire length of PVA RNA.

**Figure 5:**
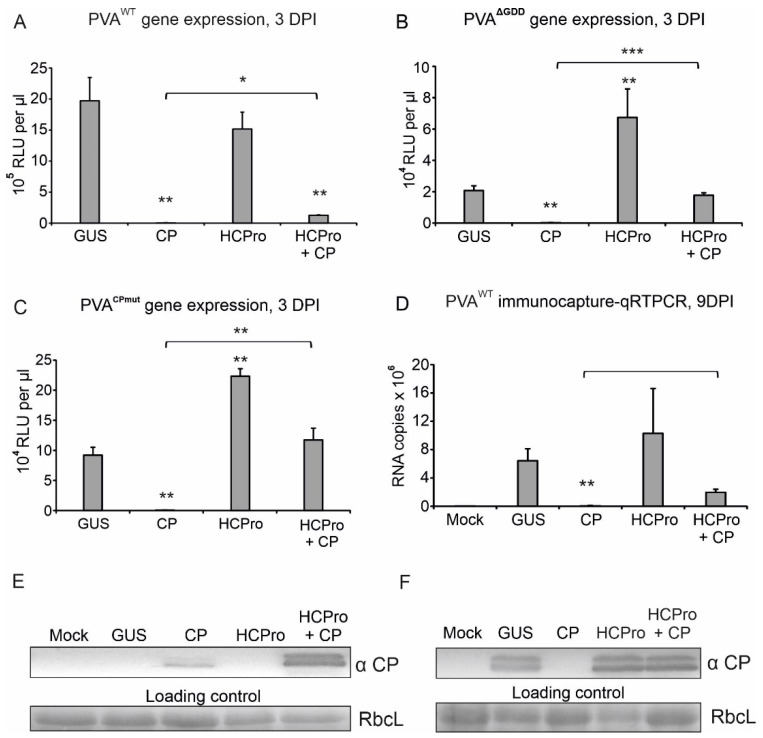
HCPro relieves the CP-mediated translational block of PVA. (**A**) PVA^WT^ was co-expressed with GUS, CP or HCPro alone or with CP and HCPro together. RLUC and CP levels were analyzed at 3 dpi. (**B**) Same as (**A**) but with the PVA^ΔGDD^. (**C**) Same as (**A**) but with PVA^CPmut^. (**D**) PVA^WT^ RNA amount from virions was measured by IC-RT-qPCR at 9 dpi in the same setup as in (**A**) *p* < 0.05 = *; *p* < 0.01 = **; *p* < 0.001 = ***. (**E**,**F**) Western blots showing CP expression in (**A**,**D**), respectively.

The next CP-inhibition experiments were carried out using PVA^WT^ and two PVA mutants, one incapable of cell-to-cell movement (PVA^CPmut^) and the other defective in replicase activity (PVA^ΔGDD^) [27]. PVA^CPmut^ and PVA^ΔGDD^ RNAs carry the *Rluc* gene between NIb and CP cistrons similarly as PVA^WT^. Followed by *Agrobacterium* infiltration of the viral constructs one day after HCPro and CP expression constructs, we measured the gene expression of PVA^WT^ at 3 dpi (Figure 5A). Again, over-expression of CP completely inhibited PVA^WT^ expression. Over-expression of HCPro had no apparent effect on RLUC accumulation from PVA^WT^ but had a significant positive effect on that from PVA^ΔGDD^ and PVA^CPmut^ (Figure 5A–C). Co-expression of HCPro and CP with the PVAs partly rescued viral gene expression from PVA^WT^ and either partly or entirely from PVA^ΔGDD^ and PVA^CPmut^, respectively, in parallel experiments (Figure 5A–C).

CP accumulation in the above-described experiments was assessed by western blot. CP overexpression could be detected in the leaf samples at 3 dpi, but not anymore at 9 dpi (Figure 5E,F). Assumably, when CP blocks viral protein production from PVA RNA, CP expression gradually ceases in the absence of the silencing suppression activity of HCPro. PVA^WT^-derived CP levels in GUS and HCPro -supplemented plants were below the detection limit at 3 dpi, whereas at 9 dpi PVA^WT^ CP was readily detectable. The double CP band, which we occasionally detect, is likely a result of proteolysis of CP in the collected samples.

To quantitate PVA particle accumulation, we subjected the samples collected at 9 dpi to IC-RT-qPCR. The amount of RNA encapsidated into virions roughly followed the pattern of viral gene expression in the GUS control, HCPro and HCPro and CP co-expressing plants at 3 dpi (compare Figure 5A,D). Instead, when an excess of transiently expressed CP was available already at the initiation of PVA infection, particle formation was practically non-existent. Based on these results we conclude that HCPro releases the vRNA from CP-mediated translational inhibition and simultaneously enables particle formation.

### 3.4. Encapsidation Occurs Both for Replicating and Non-Replicating PVA RNA but Particles Containing Non-Replicating Viral RNA Are Unstable

**Figure 6:**
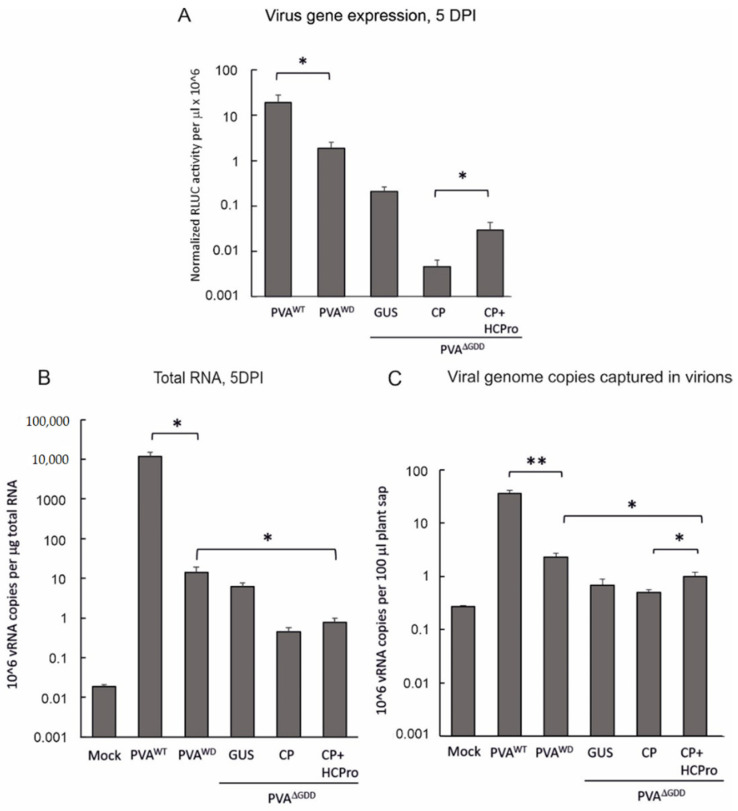
IC-RT-qPCR detects little replication-defective RNA. (**A**) PVA^WT^ and PVA^WD^ alone and PVA^ΔGDD^ together with either GUS, CP, or CP plus HCPro were transiently expressed in *N. bet-hamiana* leaves. Gene expression from PVA^WT^, PVA^WD^ and PVA^ΔGDD^ was quantitated at 5 dpi. The graph is presented in logarithmic scale to accommodate the vast differences in the gene expression amongthe mutant virus infected samples. (**B**) Quantitation of the total genomic vRNA amounts of the same samples as in (**A**). (**C**) Amount of genomic vRNA detected in the same samples as in (**B**) with IC-RT-qPCR indicating the amount of encapsidated PVA RNA. *p* < 0.01 = **, *p* < 0.05 = *.

The above experiments suggested that PVA vRNA that can engage in translation is also available for particle formation. Next, we asked whether non-replicating PVA RNA can be encapsidated into virions. To ensure high enough viral expression levels from each construct, we infiltrated *N. benthamiana* leaves with *Agrobacterium* carrying PVA^WT^ (OD_600_ 0.01), PVA^WD^ (OD_600_ 0.1) and PVA^ΔGDD^ (OD_600_ 1) constructs. We expressed PVA^ΔGDD^ RNA together with GUS as a control, CP, and CP plus HCPro (OD_600_ 0.15 + 0.15) and analyzed viral gene expression and vRNA amounts from both total RNA and immunocaptured samples at 5 dpi. The RLUC expression from PVA^ΔGDD^ was approximately 100-fold lower than PVA^WT^ (Figure 6A). For comparison, we showed the results from PVA^WD^, which expresses HCPro^WD^. PVA^WD^ replicates but cannot form stable virions [31]. PVA^WD^ produced 10-fold less RLUC than PVA^WT^ and 9-fold more RLUC than PVA^ΔGDD^. Transient expression of CP with PVA^ΔGDD^ RNA reduced the RLUC expression whereas the co-expression of HCPro with CP was able to restore it partially (Figure 6A). RLUC levels were significantly higher in CP- and HCPro-co-expressing leaves compared to CP-supplemented leaves. However, the level of HCPro-mediated restoration varied between the experiments from 10% (as in Figure 6A) to nearly 100% (as in Figure 5B), possibly depending on the ratios of available CP and HCPro. Notably, CP-mediated inhibition of PVA gene expression is concentration-dependent [14].

The RT-qPCR result revealed that one microgram of total RNA from PVA^WD^ expressing plants contained nearly 1000-fold less vRNA than that from PVA^WT^ infected plants. Furthermore, the accumulation of PVA^ΔGDD^ vRNA was even lower than PVA^WD^ RNA. When PVA^ΔGDD^ was expressed with CP, or with HCPro and CP together, the vRNA amounts did not differ significantly from each other, but they were still 100 times over the background measured from the mock sample (Figure 6B).

PVA^WT^ RNA could be detected from 100 microliters of plant sap by IC-RT-qPCR 16 times more efficiently than PVA^WD^ RNA. Thus, the relative difference between PVA^WT^ and PVA^WD^ RNA in particles was smaller than in the total RNA. The amount of immunocaptured PVA^WD^ RNA was 3 times higher than the amount of PVA^ΔGDD^ RNA. It is important to note that virion-captured PVA^ΔGDD^ RNA amounts in all PVA^ΔGDD^-containing samples were only 2–3 times higher compared to non-specific background measured from mock samples (Figure 6C), which suggests that very little particles accumulated in these plants.

**Figure 7:**
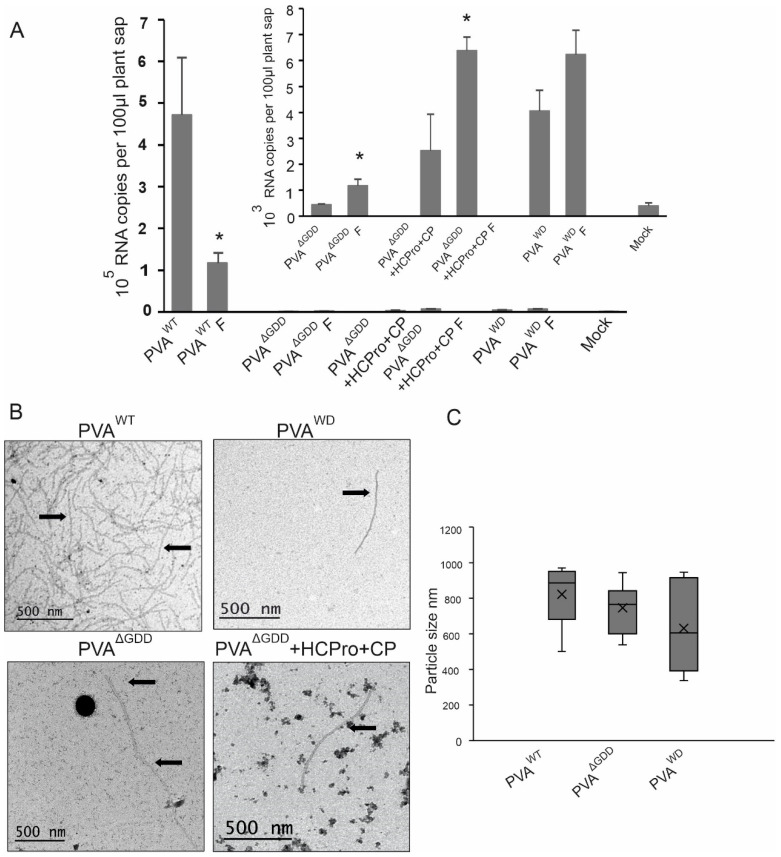
Degradation-prone particles are formed both by PVA^ΔGDD^ and PVA^WD^. (**A**) In vivo cross-linking of PVA particles. RNA copy numbers for the PVA^WT^, PVA^ΔGDD^ + HCPro + CP, PVA^ΔGDD^, PVA^WD^ detected from samples derived from non-fixed tissue and fixed tissue after reversion of the cross-link (marked by F). Student’s *t*-test between the non-fixed and fixed samples of each construct type was carried out (*p* < 0.05 = *). (**B**) PVA^WT^ particles and virus-like particles from PVA^ΔGDD^ + HCPro + CP, PVA^ΔGDD^ and PVA^WD^ visualized with negative staining and transmission electron microscopy. Arrows indicate representative particles. (**C**) Particle size measurements. Fixed PVA^WT^, PVA^ΔGDD^, PVA^WD^ particles and particle-like structures detected in TEM images were measured and the values are presented in boxplots.

We wanted to investigate even more closely if particle formation by PVA^ΔGDD^ is possible. We used formaldehyde to fix the molecular interaction between PVA RNA and CPs to protect the putative particles in the cell lysates. As in previous experiments, we used PVA^WT^ and PVA^WD^ as controls and PVA^ΔGDD^ with ectopically expressed CP and HCPro as the experimental treatments. After reversion of the cross-links in the PVA^WT^ control sample, the quantifiable PVA^WT^ RNA from the fixed tissues ranged between 12–37% of that from non-fixed tissue. This suggests that a proportion of particles remained cross-linked and was thus undetectable by the IC-RT-qPCR procedure. In the representative experiment presented in Figure 7A, the amount of quantifiable PVA RNA after reversion was around 25% of the corresponding non-fixed PVA^WT^ infected sample. Despite the incomplete reversion of cross-links, the fixed samples, PVA^WD^, PVA ^ΔGDD^ and PVA ^ΔGDD^ + CP + HCPro contained higher amount of immunocaptured PVA RNA than their non-fixed counterparts (Figure 7A). We used mock-inoculated plant samples as a negative control. vRNA detected from non-fixed PVA^ΔGDD^ was at the same level as the background measured from the mock controls. The above comparison of fixed and non-fixed samples suggests that similarly to PVA^WD^, replication-deficient PVA^ΔGDD^ RNA was encapsidated, but the resulting particles were prone to degradation when the infected cells were lysed.

Next, we checked the particles from PVA^WT^, PVA^WD^, and PVA^ΔGDD^ infections by TEM to gain visual evidence of vRNA encapsidation. For comparison, EM-grids were prepared from both non-fixed and fixed samples. We found abundant PVA^WT^ particles in plant lysates (Figure 7B). For PVA^WD^, we found more virion-like structures in the lysates of fixed samples than in the lysates of non-fixed samples. For PVA^ΔGDD^ and PVA^ΔGDD^ + CP + HCPro, we found virion-like structures in fixed samples but not in the non-fixed samples. The presence of ectopically expressed CP and HCPro in a PVA^ΔGDD^ context increased the number of virion-like structures. Consistent with the IC-RT-qPCR result, the presence of virion-like structures observed in all the mutant viruses was scarce compared to PVA^WT^. To confirm that the virion-like structures were of expected length, we measured them by using the Microscope Image Browser software version MIB1 [38]. The average size of PVA particles is 730 nm [39]. All PVA RNAs used here contain the *Rluc* gene, which increases the length of the RNA and consequently virion length by approximately by 12%. Several truncated particles were found in all samples. For quantification, we chose all virion-like structures that appeared full-length PVA virions. The box plot (Figure 7C) indicates that the lengths of the virion-like structures in PVA^ΔGDD^ and PVA^WD^ samples were nearly within the size range of PVA^WT^ particles. This suggests that the virion-like structures observed by EM most likely encapsidated PVA RNAs. Nevertheless, the little less-than-average length may indicate that PVA^ΔGDD^ and PVA^WD^ particles were more degradation-prone than those of PVA^WT^.

**Figure 8:**
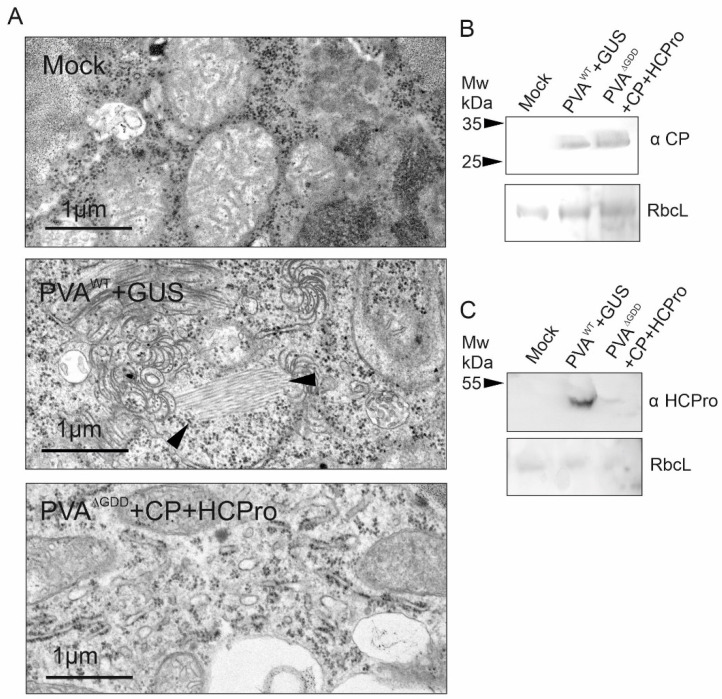
Virus particles were not detected in thin sections of PVA^ΔGDD^-infected tissues. (**A**) Virus particle stacks marked with black arrow heads were abundant in a PVA^WT^ infected leaves but were not observed in PVA^ΔGDD^ samples at 8 dpi. PVA^WT^ and PVA^ΔGDD^ were infiltrated at OD_600_ 0.1 and 0.2. PVA^WT^ was supplemented with GUS at OD_600_ 0.6 and PVA^ΔGDD^ with HCPro and CP both at OD_600_ 0.3. Samples were taken from the infiltrated leaves at 8 dpi and fixed in 2.5% glutaraldehyde prior to visualization by thin-section transmission electron microscopy. (**B**,**C**) Transient expression of CP and HCPro in PVA^ΔGDD^ + CP + HCPro leaves was confirmed by anti-CP and anti-HCPro western blots, respectively. Gel loading was checked by staining with Ponceau solution.

Next, we fixed leaf tissues of PVA^WT^ and PVA^ΔGDD^ infiltrated plants to visualize particles in thin sections of infected cells. The lower expression level of PVA^ΔGDD^ compared to PVA^WT^ was compensated for by infiltrating PVA^ΔGDD^ at 4-fold higher concentration and supplemented with both HCPro and CP. While there were abundant particle stacks and associated CI-pinwheel structures in PVA^WT^ infected leaves, none were observed in PVA^ΔGDD^ -expressing leaves (Figure 8A). The presence of transiently expressed CP and HCPro was confirmed by western blot (Figure 8B,C). According to IC-RT-qPCR the amount of immunocaptured PVA RNA in leaves expressing the non-replicating PVA^ΔGDD^ RNA was 50–100 times lower than in PVA^WT^ (see Figure 6D and Figure 7A), which may explain the difficulty in detecting particles in the thin sections of the PVA^ΔGDD^ tissues. It is also possible that the particle stacks connected to the CI-induced pinwheel structures present in PVA^WT^ panel (Figure 8A) do not form in the absence of replication.

## 4. Discussion

Potyviral HCPro functions both in RNA silencing suppression [2,3] and particle stabilization [7]. While the endogenous HCPro and a heterologous viral suppressor of gene silencing could both restore PVA^ΔHCPro^ RNA expression, CP stabilization connected to particle formation could be complemented only in the presence of PVA HCPro. As previously reported for PPV [7], this result indicates that gene silencing suppression and HCPro’s role in particle formation are distinct functions also in PVA infection. HCPro’s overexpression with PVA^WT^ RNA neither increases viral gene expression nor particle accumulation [29], indicating that enough HCPro is produced from vRNA for replication, silencing suppression and encapsidation of PVA RNA in infection.

Because PVA CP plays key roles in both the regulation of viral gene expression [14,25] and the formation of viral particles, we further investigated the interplay between HCPro and CP during these viral processes. We found that HCPro relieves CP-mediated inhibition of PVA RNA expression likely by sequestering excess CP to particles. Finally, we addressed the questions whether PVA replication is a prerequisite for encapsidation of PVA RNA into particles and present evidence for the encapsidation of PVA^ΔGDD^ RNA. The extreme instability of PVA^ΔGDD^ particles made their detection and documentation difficult. This emphasizes the essential role of replication in the formation of stable particles. From an evolutionary point of view, a packaging strategy that ensures the formation of infectious particles is logical. Restricting packaging to genomes that can be amplified increases the likelihood of further infections.

The potyviral HCPro has several functional regions located in different parts of the protein [40]. The functions related to RNA- and siRNA-binding [41], RNA silencing suppression [9], systemic movement [8,42], synergistic interaction with other viruses [4,5] and virion formation [7] are all located within the central domain between amino acids 100–300. In PPV HCPro the amino acids that contribute to virion stabilization are arginine residues 234–235 followed by a histidine residue 236 [7]. Two similarly located arginine residues (amino acids 239–240) are present in PVA HCPro. Whether these conserved amino acids contribute to PVA particle formation remains to be tested, as does studying the effect of the region surrounding these amino acids. Amino acids contributing to the long-distance movement of tobacco etch virus (TEV) have been localized to the vicinity of this region. Mutations in TEV HCPro at this site cause reduced capacity for systemic infection [42]. Although conclusive evidence is still lacking, it is nevertheless a possibility that the problems of systemic infection by TEV are due to impaired particle formation.

Many of the key issues in PVA infection center around CP functions. We have found that ectopic overexpression of CP inhibits the production of viral proteins [14,15]. This function requires the sequence encoding CP on PVA RNA [14] and CP’s capacity to bind to viral RNA [25]. This inhibition occurs separately from replication and its mechanism relates to PVA translation [14]. Although the inhibition operates through the last cistron at the 3′ end of PVA RNA, CP blocked translation throughout PVA RNA (see Figure 4). In conditions where excess CP inhibited vRNA translation and HCPro could not accumulate, encapsidation was inhibited. This is however an artificial condition, which doesn’t reflect natural PVA infection. We have earlier proposed that the translational block by CP is necessary for replication complex assembly, and its removal allows viral replication [25]. Two host chaperons and a protein kinase called CK2 maintain a low CP level in early infection [15,25]. At an appropriate stage of the infection a shift to more pronounced CP accumulation occurs. Later in the infection the higher rate of CP accumulation compared to the other viral proteins [29] indicates that more CP is produced, or it is significantly stabilized. Stabilization could be attributed to virion assembly. By the time of virion assembly, enough HCPro has accumulated to aid virion formation. In addition, a VPg-mediated virus-specific mechanism could enhance CP production at a later stage of infection [29,43]. Overall, the regulatory system must ensure the safe packaging of the vRNA into virions when the amount of CP is sufficient. HCPro is an excellent candidate for the coordination of these functions as it participates in vRNA silencing suppression [2,3], VPg-mediated enhancement of vRNA expression [43], associates with ribosomes [44,45] and is required for particle formation (see Figure 3). The results of this study suggest that HCPro can deliver CP molecules to vRNA encapsidation, thus reducing translation inhibition (see Figure 5).

Previously, no PPV particles were found in leaves expressing replication-deficient potyviral RNA [7,17]. The authors concluded that replication and particle assembly are interconnected. One possibility is that the localization of replicating vRNA in ER-bound replication vesicles could contribute to the formation of stable virus particles. In this study, detection of both immunocaptured PVA^ΔGDD^ RNA and PVA^ΔGDD^ particles was challenging (see Figure 6 and Figure 7), but covalent cross-links between CP molecules in the particles prior to homogenization of the leaf material improved the detection (Figure 7 and Figure 8). We therefore conclude that non-replicating PVA RNA could be encapsidated proposing that virion assembly would not require active replication or localization of vRNA to replication vesicles, but the resulting particles are unstable.

Considering the differences between replicating PVA^WT^ and PVA^ΔGDD^ RNA, the 5′ end structure is an obvious one. The difference in the 5′ end of replicable and non-replicating vRNA could therefore offer an alternative explanation to the role of replication in particle formation. VPg is the natural 5′ end attachment on potyvirus RNA. VPg becomes covalently linked to the 5′ end of vRNA via NIb-catalyzed VPg uridylation [46,47]. By analogy to picornaviruses [48] this is likely to be the reaction that initiates potyvirus replication. In an *Agrobacterium* infiltration-based study, PVA^ΔGDD^ RNA emerges from the nucleus presumably as a capped transcript. The 5′ cap not only protects the mRNA from degradation [49] but also facilitates translation initiation [50]. An in vitro transcript from full-length PVA cDNA is not infectious unless transcription is performed in the presence of a cap-analog [51]. Translation of RLUC from PVA^ΔGDD^ RNA demonstrates that the m7G cap is sufficient to protect PVA RNA and assist it to translation. In our previous studies, we have shown that VPg provided in trans is a powerful enhancer of PVA RNA stability and translation [29,43,52]. PVA^ΔGDD^ RNA appears to respond in the same manner as PVA^WT^ RNA to translation regulation by free cytoplasmic VPg [29,43,52]. Therefore, it is plausible that the cap can substitute VPg as the 5′ end structure in VPg-mediated regulation of PVA translation. PVA VPg binds to the eukaryotic initiation factor (iso)4E (eIF(iso)4E) [53,54]. Therefore, the ability of the cap to bind eIF4E or (iso)4E may sufficiently support virus-specific promotion of translation. In our recent study, we obtained evidence that binding of VPg-eIF(iso)4E is necessary for the stabilization of PVA RNA against RNA silencing and for delivering it to polysomes for translation [54]. PVA HCPro binds to eIF4E and eIF(iso)4E [36] and, as studied with other potyviruses, HCPro binds to VPg [55,56].

**Figure 9:**
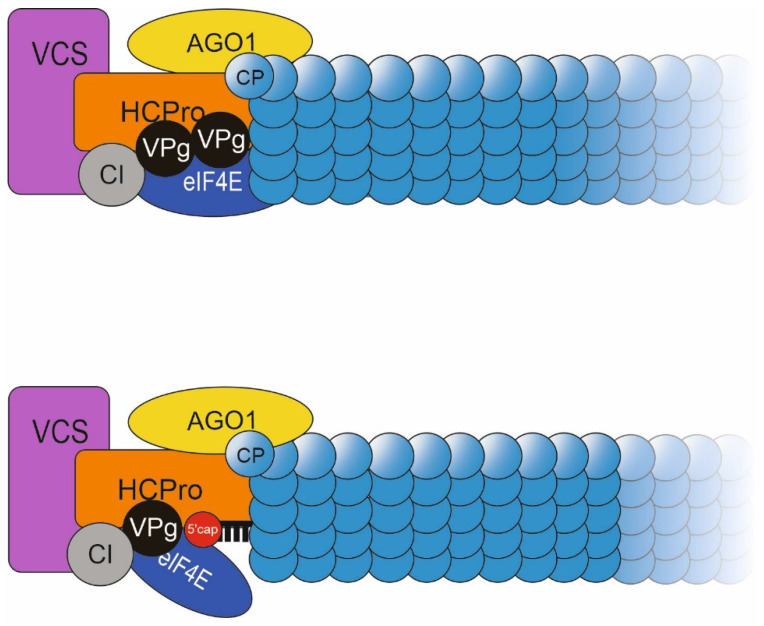
A hypothetical model for the formation of stable PVA particles. (**Top**): PVA^WT^ particle with a high-molecular weight ribonucleoprotein complex assembled around VPg covalently bound at the 5′ end of vRNA. The model incorporates known interactions between HCPro and VPg, CI, eiIF(iso)4E, VCS and AGO1 and CP. (**Bottom**): A PVA^ΔGDD^-derived particle where the m7G cap replaces the covalently-bound VPg at the 5′ end thus leaving the vRNA exposed to host RNases.

In Ivanov (2016) [44], we obtained evidence of a high molecular weight (HMW) ribonucleoprotein (RNP) complex, which we proposed assembles around the 5′ end of PVA RNA. Many of the proteins of this complex have a role in the enhanced accumulation of CP [30,31]. In a model depicted in Figure 9 we propose that this HMW RNP complex is required to ensure the packaging of replicated vRNA into stable particles. The HMW complex associates with ribosomes [30] and contains viral proteins VPg, HCPro and CI [30]. In addition, the host protein VCS is part of this complex [31]. PVA infection requires VCS, which assembles to PVA-induced granules with vRNA and is one of the components contributing to VPg-mediated translational regulation [30]. VCS is a WD40 domain-containing protein which typically acts as a scaffold in protein complexes, for example in those involved in RNA metabolism [57]. Our study with PVA HCPro^WD^ mutant provided evidence that VCS’s association with HCPro within a multiprotein complex is crucial for RNA silencing suppression, translation, particle formation and systemic spread of PVA infection [31]. Also ARGONAUTE 1 (AGO1) associates with HCPro in the HMW complex on ribosomes [44]. We recently identified a specific binding site for AGO1 in HCPro [58].

An intriguing possibility is that this large protein complex remains bound to the 5′ end of encapsidated potyviral RNA and stabilizes the particle. Atomic force microscopy has revealed that VPg, HCPro and CI reside within a large structure at the end of PVA virions encapsidating the 5′end of vRNA [22,23]. A mutation in the VCS-binding site of HCPro debilitated particle formation and systemic infection [31]. Accordingly, the lack of HCPro-VCS interaction substantially reduced the amount of particle-associated PVA^WD^ RNA (Figure 6) while cross-linking stabilized PVA^WD^ particles substantially (Figure 7). We have also demonstrated that PVA carrying a mutation interfering with the binding of AGO1 and HCPro cannot form stable particles [58]. Interestingly, the HCPro-AGO1 interaction is important for AGO1′s association with PVA CP. While VCS and AGO1 clearly have a role in the formation of stable particles, direct evidence of their association with PVA particles is still lacking.

Taken together, we propose that the m7G cap, despite its functionality in the regulation of virus-specific translation, cannot substitute VPg in virion formation. This study shows that encapsidation is possible when the interactions within the hypothetical 5′ end-linked protein complex are disturbed, but the produced particles are unstable. We suggest that proteins assembled around the 5′ end of PVA RNA seal the particles in a manner that protects the PVA genome from nucleases. Thus, in the case of non-replicating PVA^ΔGDD^ the formation of the protein complex supporting the integrity of the particles fails due to the presence of the m7G cap, and the particles become degradation-prone. A similar model could be applicable for PVY because a similar tip structure is found associated with VPg also in PVY particles [23]. In addition, eIF4E locates specifically at one extremity of lettuce mosaic virus particles [24]. The above examples suggest that that the assembly of a protective protein complex around VPg associated with vRNA in particles may be a common potyviral strategy. While another group of (+)-stranded RNA viruses, the closteroviruses, also carry a terminal structure on the particles [59], it remains to be studied whether the presented hypothesis of a protective protein complex to seal the particles applies to other virus families.

## Data Availability

Not applicable.

## References

[1] Valli A.A., Gallo A., Rodamilans B., López-Moya J.J., García J.A. (2018). The HCPro from the potyviridae family: An enviable multitasking helper component that every virus would like to have. Mol. Plant Pathol..

[2] Anandalakshmi R., Pruss G.J., Ge X., Marathe R., Mallory A.C., Smith T.H., Vance V.B. (1998). A viral suppressor of gene silencing in plants. Proc. Natl. Acad. Sci. USA.

[3] Kasschau K.D., Carrington J.C. (1998). A counterdefensive strategy of plant viruses: Suppression of posttranscriptional gene silencing. Cell.

[4] González-Jara P., Atencio F.A., Martínez-García B., Barajas D., Tenllado F., Díaz-Ruíz J.R. (2005). A single amino acid mutation in the plum pox virus helper component-proteinase gene abolishes both synergistic and RNA silencing suppression activities. Phytopathology.

[5] Shi X.M., Miller H., Verchot J., Carrington J.C., Vance V.B. (1997). Mutations in the region encoding the central domain of helper component-proteinase (HC-Pro) eliminate potato virus X/potyviral synergism. Virology.

[6] Carrington J.C., Freed D.D., Sanders T.C. (1989). Autocatalytic processing of the potyvirus helper component proteinase in Escherichia coli and in vitro. J. Virol..

[7] Valli A., Gallo A., Calvo M., de Jesús Pérez J., García J.A. (2014). A novel role of the potyviral helper component proteinase contributes to enhance the yield of viral particles. J. Virol..

[8] Cronin S., Verchot J., Haldeman-Cahill R., Schaad M.C., Carrington J.C. (1995). Long-distance movement factor: A transport function of the potyvirus helper component proteinase. Plant Cell.

[9] Kasschau K.D., Carrington J.C. (2001). Long-distance movement and replication maintenance functions correlate with silencing suppression activity of potyviral HC-Pro. Virology.

[10] Atreya C.D., Atreya P.L., Thornbury D.W., Pirone T.P. (1992). Site-directed mutations in the potyvirus HC-Pro gene affect helper component activity, virus accumulation, and symptom expression in infected tobacco plants. Virology.

[11] Atreya C.D., Pirone T.P. (1992). Mutational analysis of the helper component-proteinase gene of a potyvirus: Effects of amino acid substitutions, deletions, and gene replacement on virulence and aphid transmissibility. Proc. Natl. Acad. Sci. USA.

[12] Dolja V.V., Haldeman-Cahill R., Montgomery A.E., Vandenbosch K.A., Carrington J.C. (1995). Capsid protein determinants involved in cell-to-cell and long distance movement of tobacco etch potyvirus. Virology.

[13] Blanc S., López-Moya J.J., Wang R., García-Lampasona S., Thornbury D.W., Pirone T.P. (1997). A specific interaction between coat protein and helper component correlates with aphid transmission of a potyvirus. Virology.

[14] Besong-Ndika J., Ivanov K.I., Hafrèn A., Michon T., Mäkinen K. (2015). Cotranslational coat protein-mediated inhibition of potyviral RNA translation. J. Virol..

[15] Hafren A., Hofius D., Ronnholm G., Sonnewald U., Makinen K. (2010). HSP70 and its cochaperone CPIP promote potyvirus infection in *Nicotiana benthamiana* by regulating viral coat protein functions. Plant Cell.

[16] Andrejeva J., Puurand U., Merits A., Rabenstein F., Järvekülg L., Valkonen J.P. (1999). Potyvirus helper component-proteinase and coat protein (CP) have coordinated functions in virus-host interactions and the same CP motif affects virus transmission and accumulation. J. Gen. Virol..

[17] Gallo A., Valli A., Calvo M., García J.A. (2018). A functional link between RNA replication and virion assembly in the potyvirus. J. Virol..

[18] Anindya R., Savithri H.S. (2003). Surface-exposed amino- and carboxy-terminal residues are crucial for the initiation of assembly in Pepper vein banding virus: A flexuous rod-shaped virus. Virology.

[19] Zamora M., Méndez-López E., Agirrezabala X., Cuesta R., Lavín J.L., Sánchez-Pina M.A., Aranda M.A., Valle M. (2017). Potyvirus virion structure shows conserved protein fold and RNA binding site in ssRNA viruses. Sci. Adv..

[20] Cuesta R., Yuste-Calvo C., Gil-Cartón D., Sánchez F., Ponz F., Valle M. (2019). Structure of Turnip mosaic virus and its viral-like particles. Sci. Rep..

[21] Kežar A., Kavčič L., Polák M., Nováček J., Gutiérrez-Aguirre I., Žnidarič M.T., Coll A., Stare K., Gruden K., Ravnikar M. (2019). Structural basis for the multitasking nature of the potato virus Y coat protein. Sci. Adv..

[22] Gabrenaite-Verkhovskaya R., Andreev I.A., Kalinina N.O., Torrance L., Taliansky M.E., Mäkinen K. (2008). Cylindrical inclusion protein of potato virus A is associated with a subpopulation of particles isolated from infected plants. J. Gen. Virol..

[23] Torrance L., Andreev I.A., Gabrenaite-Verhovskaya R., Cowan G., Mäkinen K., Taliansky M.E. (2006). An unusual structure at one end of potato potyvirus particles. J. Mol. Biol..

[24] Tavert-Roudet G., Anne A., Barra A., Chovin A., Demaille C., Michon T. (2007). The potyvirus particle recruits the plant translation initiation factor eIF4E by means of the VPg covalently linked to the viral RNA. Mol. Plant Microbe Interact..

[25] Lõhmus A., Hafren A., Makinen K. (2017). Coat protein regulation by CK2, CPIP, HSP70, and CHIP is required for potato virus a replication and coat protein accumulation. J. Virol..

[26] Ivanov K.I., Puustinen P., Gabrenaite R., Vihinen H., Ronnstrand L., Valmu L., Kalkkinen N., Makinen K. (2003). Phosphorylation of the potyvirus capsid protein by protein kinase CK2 and its relevance for virus infection. Plant Cell.

[27] Eskelin K., Suntio T., Hyvarinen S., Hafren A., Makinen K. (2010). Renilla luciferase-based quantitation of potato virus a infection initiated with agrobacterium infiltration of *N. benthamiana* leaves. J. Virol Methods.

[28] Puurand U., Makinen K., Paulin L., Saarma M. (1994). The nucleotide sequence of potato virus A genomic RNA and its sequence similarities with other potyviruses. J. Gen. Virol..

[29] Saha S., Hafren A., Makinen K. (2019). Dynamics of protein accumulation from the 3’ end of viral RNA are different from those in the rest of the genome in potato virus A infection. J. Virol..

[30] Hafrén A., Lõhmus A., Mäkinen K. (2015). Formation of potato Virus A-induced RNA granules and viral translation are interrelated processes required for optimal virus accumulation. PLoS Pathog..

[31] De S., Pollari M., Varjosalo M., Makinen K. (2020). Association of host protein VARICOSE with HCPro within a multiprotein complex is crucial for RNA silencing suppression, translation, encapsidation and systemic spread of potato virus A infection. PLoS Pathog..

[32] Nakagawa T., Kurose T., Hino T., Tanaka K., Kawamukai M., Niwa Y., Toyooka K., Matsuoka K., Jinbo T., Kimura T. (2007). Development of series of gateway binary vectors, pGWBs, for realizing efficient construction of fusion genes for plant transformation. J. Biosci. Bioeng..

[33] Liu F., Grundke-Iqbal I., Iqbal K., Gong C.X. (2005). Contributions of protein phosphatases PP1, PP2A, PP2B and PP5 to the regulation of tau phosphorylation. Eur. J. Neurosci..

[34] Schindelin J., Arganda-Carreras I., Frise E., Kaynig V., Longair M., Pietzsch T., Preibisch S., Rueden C., Saalfeld S., Schmid B. (2012). Fiji: An open-source platform for biological-image analysis. Nat. Methods.

[35] Lõhmus A., Varjosalo M., Makinen K. (2016). Protein composition of 6K2-induced membrane structures formed during Potato virus A infection. Mol. Plant Pathol..

[36] Ala-Poikela M., Goytia E., Haikonen T., Rajamaki M.L., Valkonen J.P. (2011). Helper component proteinase of the genus Potyvirus is an interaction partner of translation initiation factors eIF(iso)4E and eIF4E and contains a 4E binding motif. J. Virol..

[37] Maliogka V.I., Calvo M., Carbonell A., Garcia J.A., Valli A. (2012). Heterologous RNA-silencing suppressors from both plant- and animal-infecting viruses support plum pox virus infection. J. Gen. Virol..

[38] Belevich I., Joensuu M., Kumar D., Vihinen H., Jokitalo E. (2016). Microscopy image browser: A platform for segmentation and analysis of multidimensional datasets. PLoS Biol..

[39] Bartels R. (1971). Potato virus A. CMI/AAB Description of plant viruses. Biology.

[40] Plisson C., Drucker M., Blanc S., German-Retana S., Le Gall O., Thomas D., Bron P. (2003). Structural characterization of HC-Pro, a plant virus multifunctional protein. J. Biol. Chem..

[41] Shiboleth Y.M., Haronsky E., Leibman D., Arazi T., Wassenegger M., Whitham S.A., Gaba V., Gal-On A. (2007). The conserved FRNK box in HC-Pro, a plant viral suppressor of gene silencing, is required for small RNA binding and mediates symptom development. J. Virol..

[42] Kasschau K.D., Cronin S., Carrington J.C. (1997). Genome amplification and long-distance movement functions associated with the central domain of tobacco etch potyvirus helper component-proteinase. Virology.

[43] Eskelin K., Hafrén A., Rantalainen K.I., Mäkinen K. (2011). Potyviral VPg enhances viral RNA translation and inhibits reporter mRNA translation in planta. J. Virol..

[44] Ivanov K.I., Eskelin K., Bašić M., De S., Lõhmus A., Varjosalo M., Mäkinen K. (2016). Molecular insights into the function of the viral RNA silencing suppressor HCPro. Plant J..

[45] Eskelin K., Varjosalo M., Ravantti J., Makinen K. (2019). Ribosome profiles and riboproteomes of healthy and potato virus A- and Agrobacterium-infected *Nicotiana benthamiana* plants. Mol. Plant Pathol..

[46] Puustinen P., Makinen K. (2004). Uridylylation of the potyvirus VPg by viral replicase NIb correlates with the nucleotide binding capacity of VPg. J. Biol. Chem..

[47] Anindya R., Chittori S., Savithri H.S. (2005). Tyrosine 66 of pepper vein banding virus genome-linked protein is uridylylated by RNA-dependent RNA polymerase. Virology.

[48] Rieder E., Paul A.V., Kim D.W., van Boom J.H., Wimmer E. (2000). Genetic and biochemical studies of poliovirus cis-acting replication element cre in relation to VPg Uridylylation. J. Virol..

[49] Furuichi Y., LaFiandra A., Shatkin A.J. (1977). 5′-Terminal structure and mRNA stability. Nature.

[50] Topisirovic I., Sonenberg N. (2011). Translational control by the eukaryotic ribosome. Cell.

[51] Puurand U., Valkonen J.P., Makinen K., Rabenstein F., Saarma M. (1996). Infectious in vitro transcripts from cloned cDNA of the potato A potyvirus. Virus Res..

[52] Hafren A., Eskelin K., Makinen K. (2013). Ribosomal protein P0 promotes Potato virus A infection and functions in viral translation together with VPg and eIF(iso)4E. J. Virol..

[53] Ala-Poikela M., Rajamaki M.L., Valkonen J.P.T. (2019). A Novel interaction network used by potyviruses in virus-host interactions at the protein level. Viruses.

[54] Saha S., Makinen K. (2020). Insights into the functions of eIF4E-biding motif of VPg in potato virus A infection. Viruses.

[55] Roudet-Tavert G., Michon T., Walter J., Delaunay T., Redondo E., Le Gall O. (2007). Central domain of a potyvirus VPg is involved in the interaction with the host translation initiation factor eIF4E and the viral protein HcPro. J. Gen. Virol..

[56] Yambao M.L.M., Masuta C., Nakahara K., Uyeda I. (2003). The central and C-terminal domains of VPg of Clover yellow vein virus are important for VPg-HCPro and VPg-VPg interactions. J. Gen. Virol..

[57] Xu J., Yang J.Y., Niu Q.W., Chua N.H. (2006). Arabidopsis DCP2, DCP1, and VARICOSE form a decapping complex required for postembryonic development. Plant Cell.

[58] Pollari M., De S., Wang A., Makinen K. (2020). The potyviral silencing suppressor HCPro recruits and employs host ARGONAUTE1 in pro-viral functions. PLoS Pathog..

[59] Peremyslov V.V., Andreev I.A., Prokhnevsky A.I., Duncan G.H., Taliansky M.E., Dolja V.V. (2004). Complex molecular architecture of beet yellows virus particles. Proc. Natl. Acad. Sci. USA.

